# A correlation research of Ki67 index, CT features, and risk stratification in gastrointestinal stromal tumor

**DOI:** 10.1002/cam4.1737

**Published:** 2018-08-19

**Authors:** Huali Li, Gang Ren, Rong Cai, Jian Chen, Xiangru Wu, Jianxi Zhao

**Affiliations:** ^1^ Department of Radiology Xinhua Hospital Affiliated to Shanghai Jiaotong University School of Medicine Shanghai China; ^2^ Department of Radiotherapy Ruijin Hospital Affiliated to Shanghai Jiaotong University School of Medicine Shanghai China; ^3^ Department of Pathology Xinhua Hospital Affiliated to Shanghai Jiaotong University School of Medicine Shanghai China

**Keywords:** CT, gastrointestinal stromal tumors, Ki67 index, the risk stratification

## Abstract

**Background and Objectives:**

Recurrence and metastasis are the most important factors affecting the quality of life and survival rate of patients with gastrointestinal stromal tumors (GISTs). Accurate preoperative determination of the malignant degree of GISTs and the development of a reasonable treatment plan can effectively reduce the recurrence rate. CT is currently considered the preferred imaging modality for initial assessment. Until now, there have only been a few studies investigating the relationship between CT features and recurrence of GISTs. However, the value of CT features in prognostic assessment is still unclear. In this study, we attempted to investigate the prognostic significance of CT features and the Ki67 index in GISTs.

**Methods:**

We retrospectively analyzed the clinicopathological and imaging data for 151 patients with a histopathological diagnosis of GIST who had received contrast‐enhanced CT examination and surgical resection at XinHua Hospital from October 2008 to December 2015 or Sir Run Run Shaw Hospital in 2017. Then, we explored the correlation among CT features, the Ki67 index, and risk stratification of GISTs. The correlation among CT features, the Ki67 index, and risk stratification was mainly analyzed using the Spearman rank correlation.

**Results:**

The incidence of high‐risk disease or metastasis was clearly higher in the group with Ki67 > 5% than that in the group with Ki67 ≤ 5% (*P* < 0.001). The Ki67 index was positively correlated with risk stratification (*r* = 0.558) or mitotic index (*r* = 0.619). CT imaging features including size, contour, and margin of the tumor were associated with the Ki67 index (*r* = 0.332, 0.333, and 0.302, respectively). The multivariate logistic regression analysis revealed that the tumor size [*P* = 0.043 Exp (B) = 1.150] and the presence of ulceration [*P* = 0.011, Exp (B) = 3.669] were effective variables in distinguishing between the groups with Ki67 ≤ 5% and >5%. The presence of necrosis or cystic degeneration, tumor contour, tumor margin, and pattern of enhancement were associated with risk stratification (*r* = 0.530, 0.501, 0.419, and 0.447, respectively).

**Conclusions:**

Our findings suggest that the Ki67 index is an effective complementation in predicting the prognosis of GISTs, and CT features including size, contour, and margin of the tumor, presence of necrosis or cystic degeneration, and pattern of enhancement provide evidence to support the importance of preoperative assessment.

## 1 INTRODUCTION

Gastrointestinal stromal tumors are the most common mesenchymal tumors originating in the digestive tract, with an annual incidence of 11‐19 per million.^1^ On rare occasions, they occur in extravisceral locations^2^, ^3^, ^4^ such as the omentum, mesentery, and retroperitoneum. However, even after a complete resection, a substantial proportion of patients experience disease recurrence, with an overall 5‐year survival of between 40% and 65%.^5^ Recurrence of disease after resection is predominantly intraabdominal and involves the original tumor site, peritoneum, and liver.^6^

The risk of recurrence in GISTs is admittedly related to the modified US National Institutes of Health (NIH) classification. Therefore, the prognostic factors primarily consist of the size, mitotic rate, and site (gastric or nongastric) of the primary tumor. Ki67 is a nuclear proliferation‐associated antigen. It is expressed during the growth and synthesis phases of the cell cycle but not in the G0 phase (resting phase).^7^ The prognostic value of Ki67 has been investigated in a number of studies, and its potential as a reliable marker has been shown in cancers of the breast, lung, prostate, cervix, and the central nervous system.^8^ However, the role of Ki67 in the prognostic evaluation of GISTs is uncertain. The tumor cell proliferation marker Ki67 may be a useful prognostic factor in GISTs.^9^

Accurate determination of the malignant degree of GISTs preoperatively and the development of a reasonable treatment plan is crucial to increase the survival ratio and survival quality. CT is currently considered the preferred imaging modality for the initial assessment and follow‐up for patients with GISTs.^10^ A recent study^11^ found that CT imaging features including tumor margin, size, shape, tumor growth pattern, direct organ invasion, necrosis, presence of enlarged vessels feeding or draining the mass, lymphadenopathy, and contrast enhancement pattern were associated with risk stratifications.

Therefore, in this study, we assessed the correlation between Ki67 index and risk stratification. Moreover, we attempted to determine whether there are characteristic CT features that can assist with prognostic assessment.

## 2 MATERIALS AND METHODS

### 2.1 Patients

This retrospective study was approved by the Institutional Ethics Committee of Xinhua Hospital, and the need for informed consent from the patients was waived. In our study, 151 patients with a histopathological diagnosis of GIST were included, who received contrast‐enhanced CT examination and surgical resection at Xin Hua Hospital from October 2008 to December 2015 or Sir Run Run Shaw Hospital in 2017.

### 2.2 CT acquisition

Contrast‐enhanced CT examinations were performed using one of the following MDCT scanners: Siemens Somatom Sensation 64 (Siemens, Forchheim, Germany); Philips Brilliance iCT, or Philips Brilliance 64 (Philips Medical Systems, Cleveland, OH, USA); GE LightSpeed VCT (GE Healthcare, Princeton, NJ, USA). The patients were fasted for at least 8 hours before examination. CT images were obtained during breath holding with the following parameters: 120 kV, 250 mA. The section thickness and reconstruction interval were 5.0 mm. An 80‐100 mL dose of nonionic intravenous contrast material was administered with a power injector at a rate of 3.0 mL/s. Then, at 28 and 60 seconds after injection with the agent, contrast‐enhanced scans in the arterial phase and portal venous phase were done. The CT scans were sent to a picture archiving and communication system (PACS) to be interpreted at workstations.

### 2.3 Image analysis

Abdominal CT scans of the 151 patients were read by two radiologists with 6 and 10 years of experience, who were blinded to the pathological features. The maximum size, contour, boundary, and growth pattern of the tumor with attention to the presence of ulceration, calcification, necrosis or cystic degeneration, pattern of enhancement, and enhancement degree. Each mass was assessed according to the absolute attenuation values, and the degree of enhancement in each phase of CT was measured (Hounsfield unit [Hu]). Matching elliptical regions of interest (ROIs) were placed in parenchymal areas. The CT value of the portal venous phase is usually higher than the arterial phase in GISTs. Therefore, the enhancement degree was based on the difference between the unenhanced and portal venous phase CT values of the mass. Classification of the enhancement degree was performed as follows: slight enhancement, the difference in CT value was 6‐20 Hu; moderate enhancement, the difference was 21‐40 Hu; and significant enhancement, the difference was >40 Hu.

### 2.4 Statistical analysis

SPSS version 19.0 was used to analyze the data. The methods for analysis consisted of *t* test, chi‐square test, and Spearman rank correlation. *P* < 0.05 was considered to indicate that a difference was statistically significant. At a level of significance of *P* < 0.05, multivariate logistic regression analysis was performed.

## 3 RESULTS

### 3.1 Patients

In our study, the age ranged from 9 to 86 years, and the median age was 61 years. The sex, tumor site, risk stratification, and Ki67 index of the samples are listed in Table 1. All cases without metastasis were divided into four groups according to the risk assessment table published by the NIH criteria in 2008. None of the cases had lymph node metastasis. Immunohistochemically, the Ki67 index was grouped as ≤5% and >5%. Mitotic rate (/50 HPF) was grouped as ≤5, 5‐10, and >10.

**Table 1:** Clinicopathological features of GIST patients

	No. of patients (151)	%
Sex		
Male	69	45.7
Female	82	54.3
Tumor site		
Stomach	105	69.5
Small intestine	38	25.2
Large intestine	4	2.6
Extragastrointestinal tract	4	2.6
Risk stratification		
Very low risk	10	6.6
Low risk	61	40.4
Intermediate risk	29	19.2
High risk	48	31.8
Metastasis	3	2.0
Ki‐67 labeling index		
≤5%	94	62.3
>5%	37	24.5
Unknown	20	13.2
Mitotic rate (/50 HPF)		
≤5	100	66.2
5‐10	34	22.5
>10	15	9.9
Unknown	2	1.3

### 3.2 CT features

The CT features of the 151 GISTs are summarized as follows. The mean tumor size in this study was 6.0 ± 4.7 cm. A total of 105 tumors (69.5%) had a regular outline, and 134 tumors (88.7%) were well defined. The most common growth pattern was exophytic (77/151, 51.0%). A total of 79 tumors (52.3%) demonstrated the presence of necrosis or cystic degeneration. The presence of ulceration (34/151, 22.5%) and calcification (20/151, 13.2%) was visible. One hundred and two cases (67.5%) showed heterogeneous density after enhancement. A few cases showed mucoid degeneration. The mean CT value of unenhanced scan was 33.7 ± 4.9 Hu, and it increased by 7‐106 Hu in portal venous phase; the mean difference in CT value between the unenhanced scan and portal venous phase was 41.2 ± 22.4 Hu.

### 3.3 The correlation between Ki67 index and risk stratification or mitotic index

There were no significant differences in the occurrence of groups of mitotic rate between the different tumor sites (*P* = 0.733; Table 2). The correlations between Ki67 and risk stratification or mitotic index are presented in Tables 3 and 4. The incidence of high‐risk disease or metastasis in the group with Ki67 > 5% was 78.4%, which was noticeably higher than that in the group with Ki67 ≤ 5% (13.8%). Risk stratification was significantly different between the two groups (*P* < 0.001). The Ki67 index was positively correlated with risk stratification, and the rank correlation coefficient (*r*) was 0.558. Mitotic index was significantly different between the two groups (*P* < 0.001). The Ki67 index was positively correlated with mitotic index, and the rank correlation coefficient (*r*) was 0.619.

**Table 2:** The correlation between tumor site and mitotic rate

Mitotic rate	Stomach (n = 105)	Small intestine (n = 36)	*P*‐value
≤5	73	26	0.733
5‐10	21	7	
>10	11	3	

**Table 3:** The correlation between Ki67 and risk stratification

Risk stratification	Ki67 ≤ 5% (n = 105)	Ki67 > 5% (n = 37)	*P*‐value	*r*
Very low risk	8	1	<0.001	0.558
Low risk	49	3		
Intermediate risk	24	4		
High risk or metastasis	13	29		

**Table 4:** The correlation between Ki67 and mitotic rate

Mitotic rate	Ki67 ≤ 5% (n = 94)	Ki67 > 5% (n = 36)	*P*‐value	*r*
≤5	81	8	<0.001	0.619
5‐10	10	18		
>10	3	10		

### 3.4 The correlation between CT features and the Ki67 index

The CT features between the groups with Ki67 ≤ 5% and >5% were analyzed, and then, the degree of correlation between Ki67 index and CT features was explored (Table 5). Among the analyzed CT features, size, contour, and margin of the tumor and the presence of ulceration, necrosis or cystic degeneration, and the pattern of enhancement were significantly different between the groups with Ki67 ≤ 5% and >5%. The mean tumor size was significantly greater in the group with Ki67 > 5% (8.8 ± 6.6 cm) than that in the group with Ki67 ≤ 5% (4.9 ± 3.1 cm; *P* < 0.001). The proportion of tumors with irregular contour, tumors with poor definition, the presence of ulceration, and the presence of necrosis or cystic degeneration was notably higher in the group with Ki67 > 5% (54.1%, 24.3%, 37.8%, and 73.0%, respectively) than those in the group with Ki67 ≤ 5% (21.2%, 4.3%, 12.8%, and 43.6%, respectively; *P* < 0.05). The size, contour, and margin of the tumor were relatively closely related to the Ki67 index (*r* = 0.332, 0.333, and 0.302, respectively). The presence of ulceration and the presence of necrosis or cystic degeneration had very low correlation with the Ki67 index (*r* = −0.283 and −0.265, respectively). There were no significant differences in growth pattern, pattern of enhancement, enhancement degree, and the presence of calcification between the two groups (*P* > 0.05), so the correlation analysis between the Ki67 index and these CT features did not reach statistical significance.

**Table 5:** The correlation between CT features and Ki67 index

CT features	Ki67 ≤ 5% (n = 94)	Ki67 > 5% (n = 37)	*P*‐value	*r*
Tumor size (cm)	4.9 ± 3.1	8.8 ± 6.6	<0.001	0.332
Contour				
Regular	75	17	<0.001	0.333
Irregular	19	20		
Margin				
Well‐defined	90	28	0.001	0.302
Ill‐defined	4	9		
Growth pattern				
Endophytic	29	8	0.887	0.013
Exophytic	45	24		
Mixed	17	5		
EGIST	3	0		
Ulceration	12	14	0.003	−0.283
Calcification	13	4	0.821	0.045
Necrosis or cystic degeneration	41	27	0.003	−0.265
Pattern of enhancement				
Homogeneous	35		0.101	0.150
Heterogeneous	59			
Enhancement degree				
Slight	9	7	0.538	−0.054
Moderate	46	15		
Significant	39	15		

**Figure 1:**
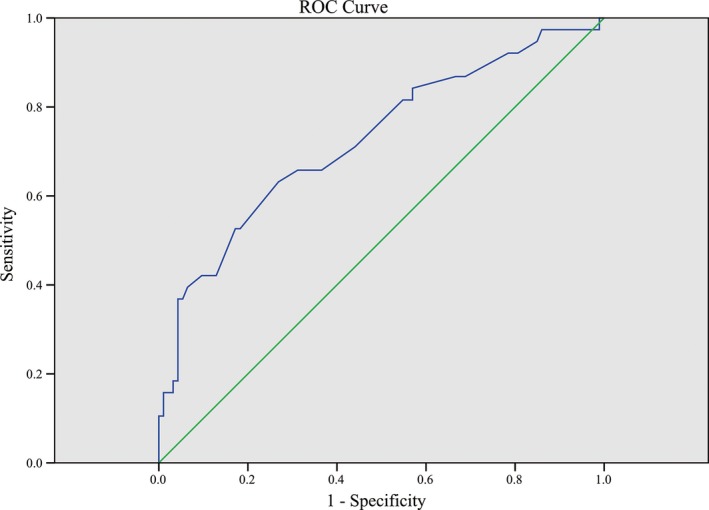
ROC curve of tumor size

The CT features with statistically significant (*P* < 0.05) in univariate analysis were included in the multivariate logistic regression analysis (Table 6). In this study, contour, margin of the tumor, and the presence of necrosis or cystic degeneration were significantly different between the two groups, but they were all demonstrated *P* > 0.05 in the multivariate logistic regression analysis. The results revealed that the effective variables in distinguishing between the groups with Ki67 ≤ 5% and >5% were tumor size [*P* = 0.043 Exp (B) = 1.150] and the presence of ulceration [*P* = 0.011, Exp (B) = 3.669]. The best cut‐off value of tumor size was analyzed using the ROC curve (Table 7, Figure 1), the area under the ROC curve (AUC) was 0.726, and the cut‐off point was 5.75 cm.

**Table 6:** Logistic regression analysis of related factors of Ki67 index in GISTs

	*P*	OR	95% CI
Tumor size (cm)	0.043	1.150	1.004‐1.317
Ulceration	0.011	3.669	1.355‐9.929

**Table 7:** Parameters of ROC curves

	AUC	SE	*P*	95% CI	Cut‐off point
Tumor size (cm)	0.726	0.051	<0.001	0.626‐0.827	5.75

### 3.5 The correlation between CT features and risk stratification

**Figure 2:**
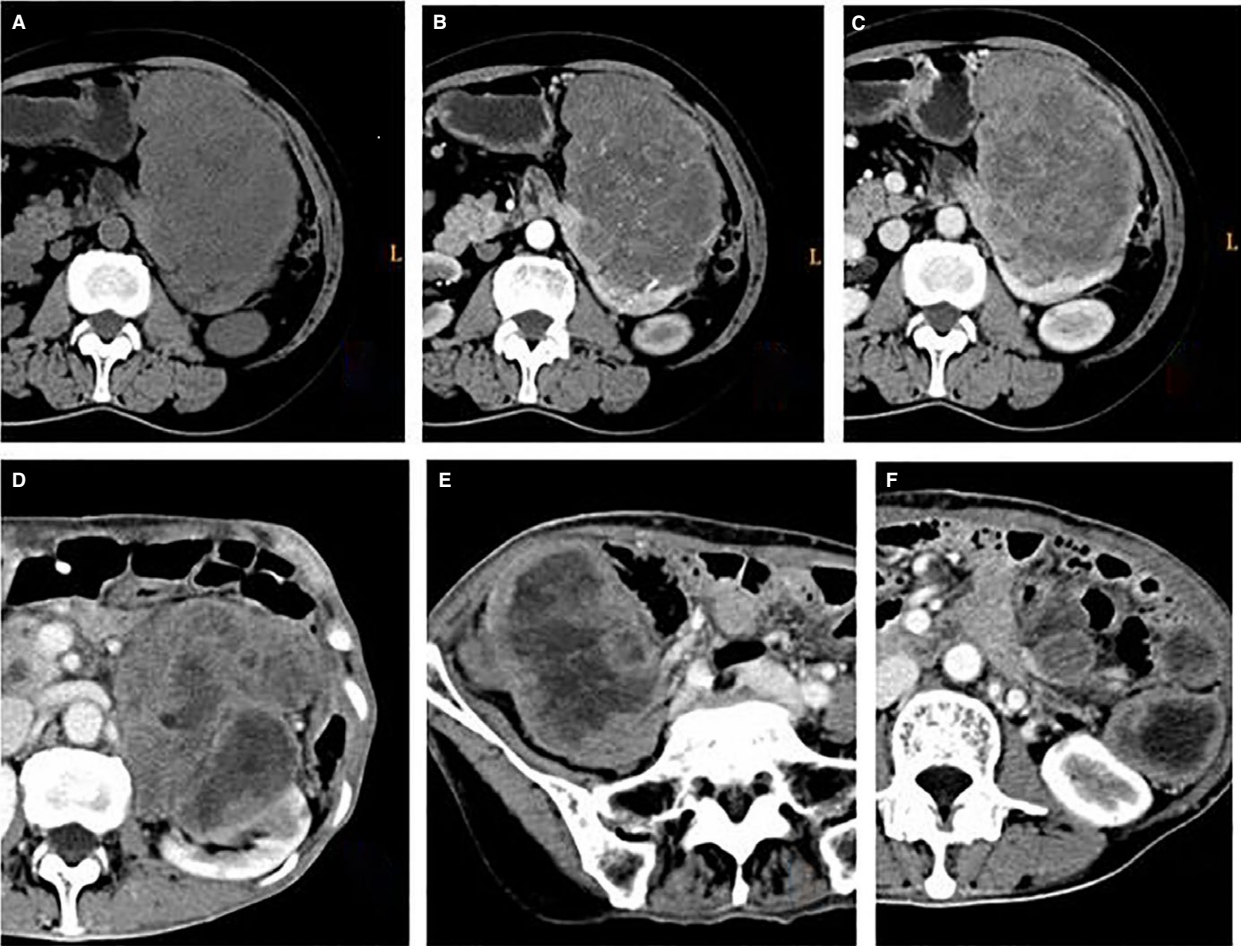
A 67‐y‐old woman with a exophytic GIST in the stomach, high risk. Unenhanced and enhanced CT (A‐C) shows a large, irregular, ill‐defined mass with necrosis and heterogeneous enhancement. Nine months after resection, enhanced CT (D‐F) demonstrates the multiple intraperitoneal recurrence and metastases

**Figure 3:**
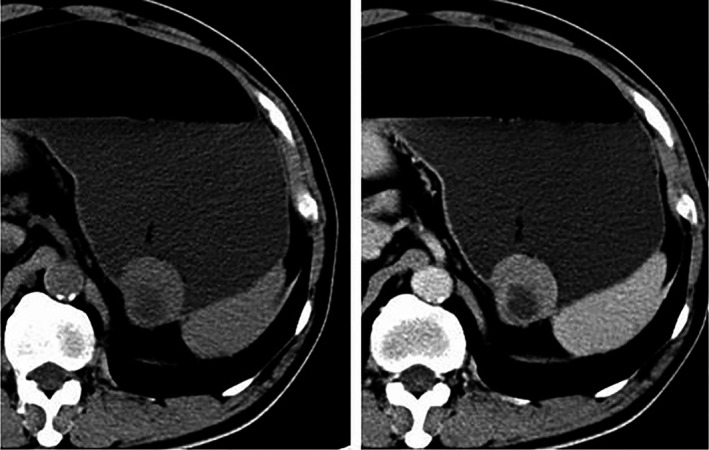
A 56‐y‐old man with a GIST in the stomach, low risk. Unenhanced and enhanced CT shows a 4‐cm mass with regular contour, well defined, necrosis, and moderate enhancement

The CT features between different risk stratification groups were analyzed, and then, the degree of correlation between CT features and risk stratification was explored (Table 8; Figures 2 and 3). Among the analyzed CT features, contour, margin, ulceration, and growth pattern of the tumor and the presence of necrosis or cystic degeneration, and pattern of enhancement were significantly different between the different risk stratification groups (*P* < 0.05). The contour and the presence of necrosis or cystic degeneration were closely related to risk stratification (*r* = 0.501 and 0.530, respectively). The margin of the tumor and the pattern of enhancement were correlated to a lower extent to risk stratification (*r* = 0.419 and 0.447, respectively). The presence of ulceration and the growth pattern of the tumor exhibited weak correlation with risk stratification (*r* = −0.170 and 0.201, respectively). There were no significant differences in the presence of calcification and enhancement degree between the different risk stratification groups (*P* ≥ 0.05), Therefore, the correlation analysis between risk stratification and these CT features did not reach statistical significance.

**Table 8:** The correlation between CT features and risk stratification

CT Features	Risk stratification				*P*‐value	*r*
	Very low (n = 10)	Low (n = 61)	Intermediate (n = 29)	High risk or metastasis (n = 51)		
Contour						
Regular	9	55	23		<0.001	0.501
Irregular	1	6	6	33		
Margin						
Well‐defined	10	61	29		<0.001	0.419
Ill‐defined	0	0	0	16		
Growth pattern						
Endophytic	6	24	11		0.013	0.201
Exophytic	2	25	16	34		
Mixed	2	11	2	7		
EGIST	0	1	0	3		
Ulceration						
Yes	0	12	6	16	0.037	−0.170
No	10	49	23	35		
Calcification						
Yes	1	8	3	8	0.645	−0.038
No	9	53	26	43		
Necrosis or cystic degeneration						
No	10	42	13	8	<0.001	0.530
Yes	0	19	17	43		
Pattern of enhancement						
Homogeneous	8	29			<0.001	0.447
Heterogeneous	2	32	21	47		
Enhancement degree						
Slight	2	5	3	9	0.050	−0.162
Moderate	3	25	21	24		
Significant	5	31	5	18		

## 4 DISCUSSION

In this study, the incidence of high‐risk disease or metastasis was noticeably higher in the group with Ki67 > 5% than that in the group with Ki67 ≤ 5% (*P* < 0.001). The Ki67 index was positively correlated with risk stratification(*r* = 0.558) or mitotic index (*r* = 0.619). Furthermore, our results demonstrated that the CT imaging features including size, contour, and margin of the tumor were associated with the Ki67 index (*r* = 0.332, 0.333, and 0.302, respectively). The multivariate logistic regression analysis revealed that the tumor size [*P* = 0.043 Exp (B) = 1.150] and the presence of ulceration [*P* = 0.011, Exp (B) = 3.669] were effective variables in distinguishing between the groups with Ki67 ≤ 5% and >5%. In addition, the presence of necrosis or cystic degeneration, tumor contour, tumor margin, and pattern of enhancement were associated with risk stratification (*r* = 0.530, 0.501, 0.419, and 0.447, respectively).

Although the Ki67 index is an important immunohistochemical marker of proliferation in tumors, its prognostic value and related predictive ability in GISTs have not been well established. There have been a number of studies trying to investigate the value of Ki67 in the evaluation of prognosis in recent years. Zhao et al^12^ reported that the Ki67 index (≤5, 5‐8, and >8%) was an independent predictor related to recurrence‐free survival of GIST patients; a Ki67 index >8% can supplement the modified NIH criteria for distinguishing different outcomes in high‐risk GIST patients and unfavorable response to imatinib adjuvant therapy. Turkel Kucukmetin et al^13^ identified a high Ki67 index (≥10%) as an independent predictor of both poor overall survival and poor disease‐free survival. Belev et al^14^ showed that the cut‐off value of 6% was statistically significant in terms of relapse and concluded that Ki67 was a significant prognostic factor for GIST recurrence, which could be of great importance in evaluating the malignant potential of the disease. Our results demonstrated that the incidence of high‐risk disease or metastasis was clearly higher in the group with Ki67 >5% than in the group with Ki67 ≤ 5% (*P* < 0.001). And the mitotic index was higher in the group with Ki67 >5% than in the group with Ki67 ≤ 5% (*P* < 0.001). The Ki67 index was positively correlated with risk stratification (*r* = 0.558) or mitotic index (*r* = 0.619). Therefore, high Ki67 positivity seems to be an important finding for clinical follow‐up and management of disease. Then, we discussed the correlation between CT features and Ki67 index to determine the prognostic value of CT features.

CT, with its panoramic capabilities and high‐contrast resolution, provides essential information for treatment planning and for the follow‐up of GIST patients treated with surgery or chemotherapy.^15^ The risk stratification of gastric GISTs is currently based on the size of tumor and mitotic count. Large tumor size is a known risk factor for GIST. In addition, tumor size has been determined as the most important factor for recurrence in gastric GIST patients who underwent radical resection.^16^ A previous study^17^ on 143 patients with gastric GIST documented that tumor size >10 cm, irregular/lobulated outline, and presence of an enhancing solid component were independent predictors of metastatic disease. However, the conclusion that the presence of an enhancing solid component (defined as a solid component >1 cm with enhancement beyond the psoas muscle) was an independent predictor of metastatic disease was different from ours. In our study, there was no significant difference in the enhancement degree between the different risk stratification groups (*P* > 0.05), and there was no correlation between enhancement degree and risk stratification. However, enhancement degree plays an important role in distinguishing GISTs from other tumors such as leiomyomas.^18^ In our study, large tumor size, the presence of necrosis or cystic degeneration, irregular outline, and ill‐defined or heterogeneous enhancement indicated high‐risk GIST. However, in some cases, magnetic resonance imaging (MRI) and positron‐emission tomography (PET) combined with CT may be useful for predicting the malignant potential of GISTs.^19^, ^20^ This issue should be further explored later.

There are some limitations of the present study. First, it is a retrospective review of imaging with a limited number of patients. In addition, we did not have information on whether the patient experienced recurrence or death, due to the lack of long‐term follow‐up.

In conclusion, the Ki67 index is an important complement in evaluating the prognosis of GISTs. The size, contour, and margin of the tumor and the presence of necrosis or cystic degeneration, and the pattern of enhancement provide important information for assessing the prognosis before surgery and to help determine the clinical treatment plan. These findings should be validated in larger studies in the future.

## CONFLICT OF INTEREST

None declared.
